# Efficacy of antibacterial agents combined with erbium laser and photodynamic therapy in reducing titanium biofilm vitality: an in vitro study

**DOI:** 10.1186/s12903-023-02730-8

**Published:** 2023-01-19

**Authors:** Jing Wang, Tengyu Geng, Yuzhuo Wang, Changyong Yuan, Penglai Wang

**Affiliations:** 1 School of Stomatology, Xuzhou Medical University, Xuzhou, China; 2 Center of Implant Dentistry, Affiliated Stomatological Hospital of Xuzhou Medical University, Xuzhou, China; 3 Department of Prosthodontics, Affiliated Stomatological Hospital of Xuzhou Medical University, Xuzhou, China

**Keywords:** Biofilms, Dental implants, Peri-implantitis, Laser therapy, Photodynamic therapy

## Abstract

**Background and objective:**

The emergence of peri-implant diseases has prompted various methods for decontaminating the implant surface. This study compared the effectiveness of three different approaches, chlorhexidine digluconate (CHX) combined with erbium-doped yttrium–aluminum–garnet (Er:YAG) laser, photodynamic therapy (PDT), and CHX only, for reducing biofilm vitality from implant-like titanium surfaces.

**Study design/Materials and methods:**

The study involved eight volunteers, each receiving a custom mouth device containing eight titanium discs. The volunteers were requested to wear the device for 72 h for biofilm development. Fluorescence microscopy was used to evaluate the remaining biofilm with a two-component nucleic acid dye kit. The vital residual biofilm was quantified as a percentage of the surface area using image analysis software. Sixty-four titanium discs were assigned randomly to one of four treatment groups.

**Results:**

The percentage of titanium disc area covered by vital residual biofilm was 43.9% (7.7%), 32.2% (7.0%), 56.6% (3.6%), and 73.2% (7.8%) in the PDT, Er:YAG, CHX, and control groups, respectively (mean (SD)). Compared to the control group, the treatment groups showed significant differences in the area covered by residual biofilm (*P* < 0.001). CHX combined with Er:YAG laser treatment was superior to CHX combined with PDT, and CHX only was better than the control.

**Conclusion:**

Within the current in vitro model's limitations, CHX combined with Er:YAG laser treatment is a valid method to reduce biofilm vitality on titanium discs.

## Introduction

Peri-implant diseases have increased in recent years since implant dentures are becoming a highly demanded clinical treatment. French et al. found that the 8-and 10-year incidences of peri-implant mucositis and peri-implantitis were 11.9% and 7.1% [1]. Peri-implantitis involves peri-implant mucosal inflammation and associated peri-implant bone loss caused by plaque biofilm colonization on the implant surface [2, 3]. The rough thread structure on the implant surface promotes osseointegration while creating conditions favourable for plaque colonization [4]. It was reported that the sequence of microbial colonization around implants is similar to periodontitis. There is a close relationship between the flora of the "red complex" and peri-implant inflammation [5]. Further, Pontoriero et al. established a causal relationship between plaque accumulation and disease onset [6].

Treatment of peri-implantitis mainly relies on eliminating plaque biofilm from the implant surface [7]. Conventional implant surface cleansing includes the use of normal curettes [8], ultrasonic scalers [9], and air-flow abrasion [10]. However, the effectiveness of these treatment modalities is limited in some clinical scenarios. For example, some implant areas are inaccessible due to the devices' low angle and limited working distance and implant microphotography, leading to microbial colonization and biofilm formation [11]. Adjunctive therapies, including low-level laser therapy and antimicrobial photodynamic therapy (PDT), are being investigated to promote the effectiveness on peri-implantitis.

The Er:YAG laser, with a wavelength of 2940 nm, can absorb water from plaque microbes and rapidly vapourize it, resulting in micro-bursting and bacteria death [12–14]. Er:YAG laser light energy, ranging from 20 to 50 mJ, 40–55 Hz, can effectively eliminate bacterial biofilms [15]. In an animal study, Schwarz et al. found that the Er:YAG laser promoted more osseointegration than ultrasonics or plastic curets [16]. Furthermore, Nevins et al. reported that surgical therapy with Er:YAG laser reduced gingival inflammation, improved bone-implant contact, and generated new bone [17].

PDT treatment for peri-implant diseases is another option [18]. PDT stains bacteria with photosensitizer molecules and effectively kills various microorganisms when combined with a low-intensity laser [19]. Theoretically, absorption of a light source at a specific wavelength by a photosensitizer triggers a series of reactions, during which the photosensitizer leaps from the ground state to the triplet state, producing monomorphic oxygen and reactive Oxygen Species (ROS) [20]. Monomorphic oxygen attacks the surrounding biomolecules and disrupts bacterial cell walls and membranes, thus disrupting bacterial metabolism. Monomorphic oxygen has a short life cycle and acts only at the site of the lesion without affecting distant molecules, cells, and tissues [21]. Chemical compounds, such as toluidine blue (TBO), indocyanine green (ICG), and methylene blue (MB), are used for PDT [22].

Peri-implantitis is also clinically treated with adjuvant antimicrobial agents to eliminate the causative organisms. The antibacterial drugs to treat peri-implantitis commonly include 0.12–0.2% chlorhexidine digluconate (CHX) [23], 3% hydrogen peroxide (H_2_O_2_) [24], etc. CHX, both solution and gel, assists peri-implantitis treatment and reduces bacterial adhesion to tooth surfaces by the synergistic action of glycoproteins in saliva [25].

A quantitative assessment of therapeutic approaches is of great importance, given the absence of widely accepted treatment for peri-implantitis. The primary objective of this clinical in vitro trial was to compare the efficacy of Er:YAG laser, PDT, and CHX in reducing biofilm vitality from dental implant-like titanium surfaces. The hypotheses are (1) Er:YAG laser and PDT are more effective in reducing biofilm vitality than CHX and (2) Er:YAG laser is more effective in reducing biofilm vitality than PDT in residual biofilms.

## Materials and methods

### Study population

Eight healthy volunteers (four males and four females, age 29 to 34) were recruited in this study. This clinical study was approved by the ethics committee of the Affiliated Stomatological Hospital of Xuzhou Medical University (2021–003). The investigation was registered in the Chinese Clinical Trials Registry (ChiCTR2100054803). All participants in the study signed the written consent forms. Inclusion criteria were as follows: (1) adults (aged ≥ 18 years); (2) have sufficient teeth to wear an occlusal device; (3) clinically periodontal health, and gingival health (< 10% bleeding sites, probing depths ≤ 3 mm [26]). Exclusion criteria were as follows: (1) multiple tooth loss; (2) inability to wear an occlusal appliance due to chronic obstructive pulmonary disease (COPD) or sleep apnea; (3) chronic periodontitis, debilitating or uncontrolled medical conditions; (4) smoking, pregnancy; (5) allergies to acrylic or titanium; (6) recent use of antibiotics.

### Study procedures

#### Clinical examination, consent and device preparation

Medical and demographic data were collected from each study participant. Periodontal examination included probing depth, gingival recession, attachment level, and bleeding on probing (BOP). If the subject conformed to the study criteria, alginate impression materials and plastic trays were be used to make maxillary models of subjects.

In the following visits, plastic maxillary casts were prepared according to the standard procedure. Eight titanium pieces (5 mm in diameter, 1 mm thick, acid-etched, sandblasted, Wego, Shandong, China) were bonded to the buccal surfaces of the plaster model's canine, premolars, and first molars. The occlusal device was made by a technician using a 1 mm-diaphragm (Erkodur, Germany) through a pressure thermal-molding machine (ERKO-FORM-3, Erkodur, Germany) covering the attached discs. In this step, the occlusal device was removed from the plaster model and cut into the desired shape with smoothly polished edges. The new titanium discs of the same size and count were glued to the inner surface of the occlusal device using a resinoid bond with 1 mm distance to the teeth. Removing device influence could be excluded by maintaining this distance while ensuring a moist and nutritious environment [27]. 0.12% CHX was used to disinfect the occlusal device (Chenpai, Haimen, China).

#### Use of the occlusal devices

**Fig. 1:**
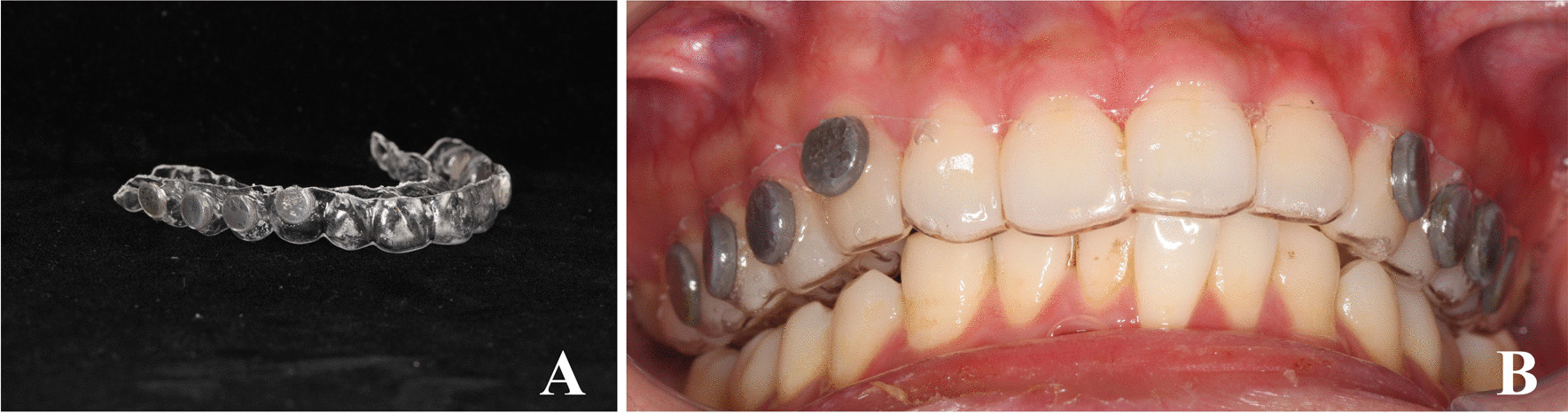
**A** Oral titanium sheet device; **B** Subject wearing the study appliance with titanium discs attached

Study participants were instructed to wear the titanium disc device (Fig. 1) for 72 h except during meals. During this period, the individual was not permitted to clear his teeth with dental floss or toothpaste. Mouthwash was not allowed either. The device was placed in saline to keep the surface of the titanium discs moist when it was removed during meals.

#### Device retrieval and analysis

The devices were collected after 72 h, and immediately subjected to following treatments to determine which method is most effective in reducing the titanium surface biofilm vitality.

It was important not to damage the surface biofilm when removing the titanium discs from the appliance. Intraoral locations of the discs were recorded and numbered. Discs retrieved from each occlusal appliance were randomized into the four treatment groups according to the computerized randomization list. Each subsequent experimental treatment was carried out for 1 min:

1. PDT Group
  The titanium discs were first treated with CHX for 1 min and then cleaned with phosphate buffered saline (PBS). The surface of the titanium discs were then injected with 100 μL of TBO photosensitizer (0.01%, Periowave, Zhengzhou, China) under light-protected conditions and dark-treated for 1 min. The PDT therapy (HHL-1000, Periowave, Zhengzhou, China) was performed by irradiating with a laser wavelength of 660 nm and output power of 2 mW vertically over the titanium disc for 1 min.
2. Er:YAG Group
  The titanium discs were first treated with CHX and then cleaned with PBS, irradiated using an erbium laser (Light Walker, Fotona, Slovenia) with a laser wavelength of 2940 nm, with an energy setting of 50 mJ/p, a frequency of 30 Hz and air/water spray at 4/4, and with the tip approximately 1 cm from the surface of the titanium discs, without contact and overlapping linear movement.
3. CHX Group
  The titanium discs were placed in CHX for 1 min, followed by rinsing off the residual CHX with PBS.
4. Control Group
  The control samples were left untreated. A single person was assigned to carry out all titanium disc handling operations for consistency.

After treatment, titanium discs were treated with a Live/Dead Nucleic Acid dye (LIVE/DEAD™ BacLight™ Bacterial Viability Kit, Thermo Fisher Scientific, USA). Live bacteria were stained with SYTO9 (green), while dead bacteria were stained with PrI (red). After gently washing with PBS, a 500 mix of staining solution was added to each culture dish and incubated for 15 min at room temperature in darkness.

The stained titanium slices were observed using confocal laser scanning microscopy (CLSM, STELLARIS 5, Leica, Germany). Each titanium slice was viewed with an argon laser (514–488 nm) and a helium–neon laser (543 nm) at three random fields.

### Image analysis

The fluorescence intensity of each image was measured using ImageJ software (NIH, Bethesda, USA). The total bacterial biomass was expressed as the mean fluorescence intensity for live and dead staining. The percentage of live bacteria present was calculated by multiplying the fluorescence intensity units of live (SYTO9) staining by the standard fluorescence intensity units of live and dead staining.

### Statistical analysis

The group computed descriptive statistics (means, SDs, minima, and maxima). The Shapiro–Wilk test was used to determine the normality of the data. Since non-normal distributions were assumed to occur, we analyzed data by applying non-parametric Kruskal–Wallis test for differences in treatments. Post hoc analysis with the Mann–Whitney U test was performed using Bonferroni correction to test the differences between groups. *P* < 0.05 was considered statistically significant. SPSS (Version 20, IBM, USA) was used for the analysis.

## Results

### Study subjects

Eight subjects and 64 titanium discs were in total, while each group contained 16 discs. No adverse effects were observed in the study. Thus, all titanium tablets were included in the later studies of this trial.

### Biofilm formation on titanium surfaces

**Fig. 2:**
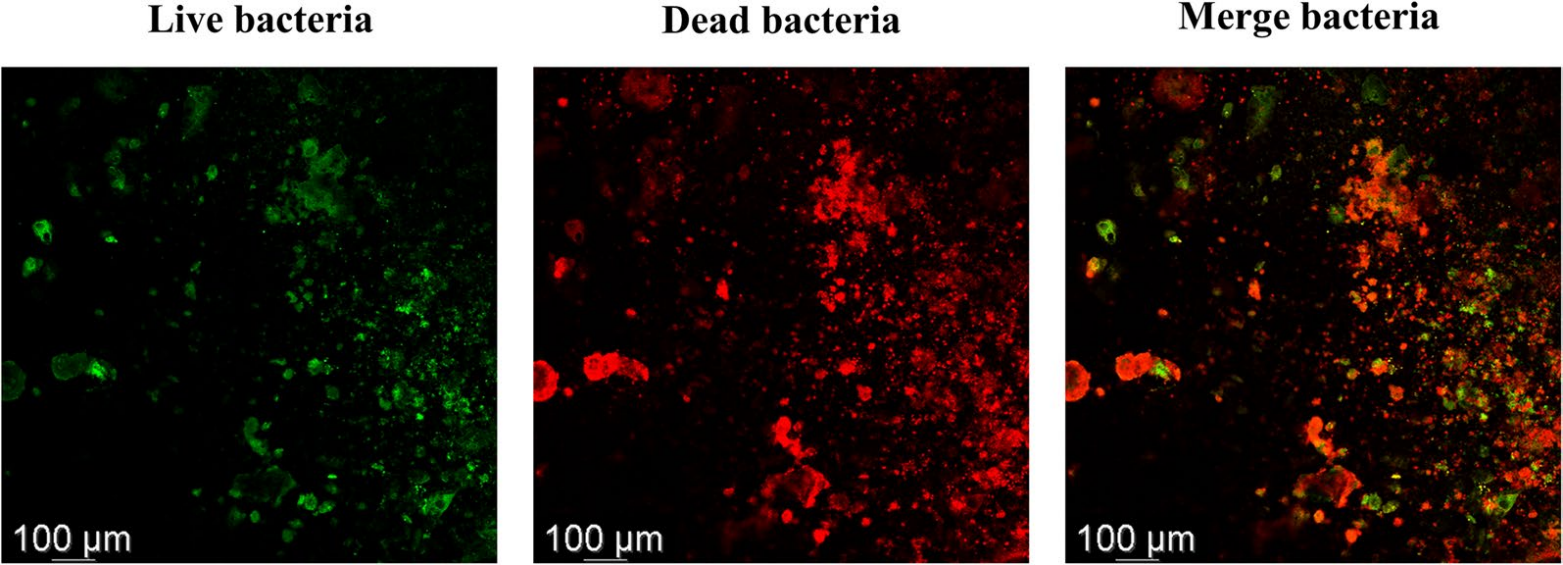
Biofilm on SLA titanium surface with fluorescent microscopic images. Fluorescence microscopy was used to visualise multispecies biofilms stained with live-dead fluorescent dyes (magnification × 10). Green staining—live bacteria only; red staining—dead bacteria only; yellow—live and dead bacteria

In a typical titanium biofilm, non-confluent clusters of bacteria were surrounded by the area where bacteria were poorly dispersed (merge image, Fig. 2). The biofilm that made up the clusters was made up of living bacteria (green staining, Fig. 2), while some clusters also had dead bacteria (red or yellow staining, Fig. 2).

### Reduction of biofilm vitality with various clinical disinfection methods

**Fig. 3:**
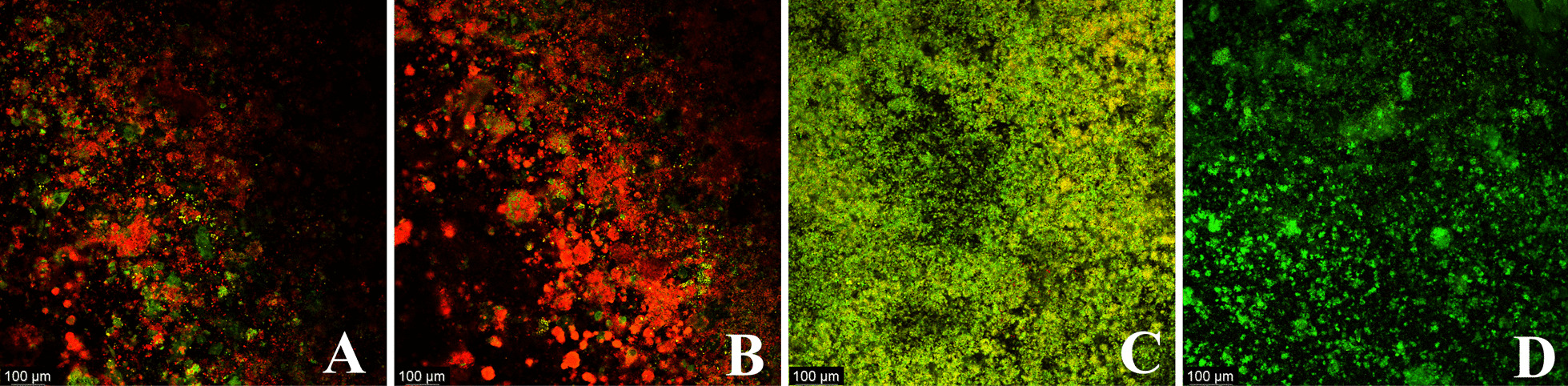
Representative images of Ti (Titanium) discs that were worn by subjects for 3 days and then stained (magnification × 10). The panels show: **A** PDT; **B** Er:YAG; **C** CHX; **D** Control

**Fig. 4:**
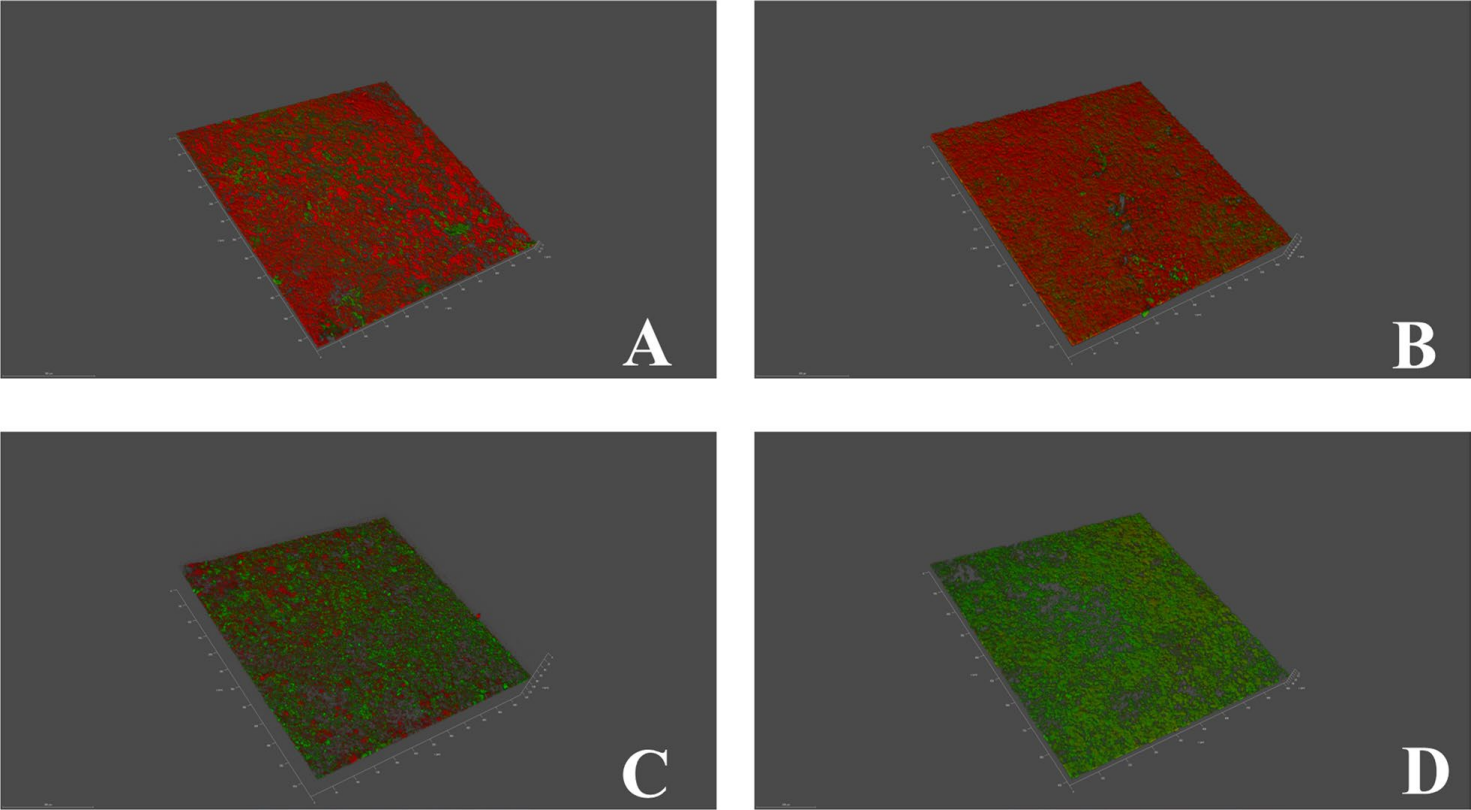
3-dimensional structure of biofilms on titanium discs. The panels show: **A** PDT; **B** Er:YAG; **C** CHX; **D** Control

The efficacy of commonly used methods for biofilm vitality reduction methods was evaluated: CHX, PDT, and Er:YAG laser. The percentage of viable plaque biofilm remaining on the surface of the titanium sheets was 21.8–59.3% (43.9% (7.7%)), 16.7–43.4% (32.2% (7.0%)), 52.9–69.2% (56.6% (3.6%)), and 63.2–85.6% (73.2% (7.8%)) in the PDT, Er:YAG, CHX, and control groups, respectively. Table 1 shows descriptive statistical information for the remaining live plaque biofilms. Figures 3 and 4 display examples of 2D fluorescence intensity and 3D biofilm images.

**Table 1:** Biofilm-covered area (%) with SYTO 9 staining

	Mean(SD)	Minium	Maximum	S^2^
PDT	43.9 (7.7)	21.8	59.3	60.4
Er:YAG	32.2 (7.0)	16.7	43.4	49.0
CHX	56.6 (3.6)	52.9	69.2	13.1
Control	73.2 (7.8)	63.2	85.6	61.4

The data in this study were non-normally distributed (*P* < 0.05), and all four groups were statistically different from each other (*P* < 0.001). All combinations were effective in reducing the biofilm vitality compared to negative controls (*P* < 0.001), and the differences in the area of biofilm vitality reduction between groups were statistically significant (Er:YAG/PDT *P* = 0.025; Er:YAG/CHX *P* < 0.001, PDT/CHX *P* = 0.005).

**Fig. 5:**
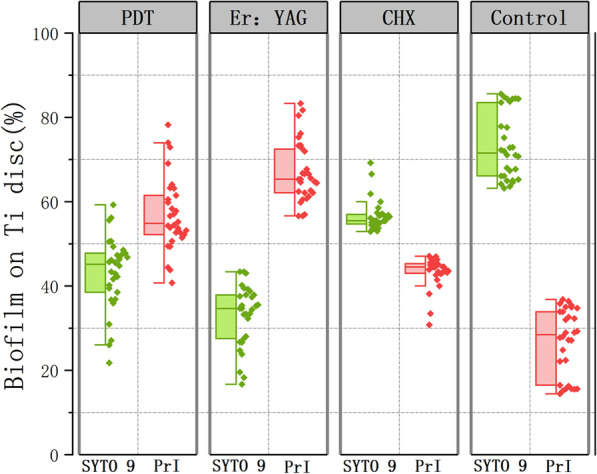
Grouped Half Box Plot plots depicting analyses of SYTO 9 and Prl stained images

Similar to the SYTO9 images, 64 PrI images were accessible for analysis. The percentage of dead plaque biofilm coverage on the surface of the titanium sheets as determined by the PrI signal was 40.7–78.2% (56.6% (8.4%)), 56.6–83.3% (67.3% (7.1%)), 30.8–47.1% (43.4% (3.6%)), 14.4–36.8% (26.9% (7.9%)), in the PDT, Er:YAG, CHX, and control groups. The data were non-normally distributed (*P* < 0.05), similar to the SYTO9 files. Four groups in the dead biofilm showed statistically significant differences between groups (*P* < 0.001). Table 2 shows statistical information for the remaining dead plaque biofilms. Figure 5 displays grouping box diagram analyses of the SYTO9 and PrI staining images.

**Table 2:** Biofilm-covered area (%) with PrI staining

	Mean(SD)	Minium	Maximum	S^2^
PDT	56.5 (8.4)	40.7	78.2	70.0
Er:YAG	67.3 (7.1)	56.6	83.3	50.0
CHX	43.4 (3.6)	30.8	47.1	13.1
Control	26.8 (7.9)	14.4	36.8	62.5

## Discussion

This study aimed to compare the residual biofilm vitality on titanium discs after plaque removal with CHX combined with Er:YAG laser and PDT. The Er:YAG, PDT, and CHX treatments were more effective in reducing biofilm vitality than the control treatment. The results indicated that the CHX combined with Er:YAG could effectively reduce the biofilm vitality from the titanium disc surface.

Peri-implantitis is a complicated clinical condition with diverse microbiota [3]. From this standpoint, determining which method is most effective against the microorganisms involved in the clinical condition of peri-implantitis is extremely important in developing treatment solutions for the disease. In contrast to previous studies that cultured mono sepcies biofilms on the titanium piece [28], we developed biofilms inside the subjects' mouths to simulate the peri-implant polymicrobial environment.

According to the literature, studies of polymicrobial infections can benefit from using in vitro models of biofilms containing multiple species [29–31]. Desch's experimental plaque model demonstrated that subjects wearing oral appliances for 72 h could develop a plaque biofilm on the titanium surface with a microbial composition similar to that of the microbiota surrounding healthy implants [30]. Unlike Desch's experimental [30], we followed Hani S's model of an oral titanium device with plaque incubated for 72 h and a 1 mm gap between the titanium piece and the tooth surface [29]. This in *vivo* model is more comfortable for the subject to wear and reduces the probability of the titanium piece being dislodged. The difference between our approach and Hani S's study was that we used CLMS to observe the 3D images of the plaque biofilm. In addition, studies have leveraged colony-forming unit (CFU) counts to analyze the biofilms on titanium plaque surfaces [32, 33]. Regarding quantitative and qualitative analysis, CFU count is less sensitive since it is vulnerable to user bias and counting error [34]. One recent study used fluorescent dye-based CLMS, which allows for more precise quantification and analysis of biofilm content and simultaneous evaluation of both living and dead microorganisms. The evaluation method is beneficial because dead and viable microorganisms contribute to the virulence of a biofilm and should not be overlooked [35].

The vital residual biofilm area was reduced by all of the decontamination methods used in this study (*P* < 0.05). Er:YAG demonstrated the best efficiency in reducing biofilm vitality on a flat titanium surface, although the effectiveness of Er:YAG on the thread-like surface of implants remains controversial. Due to the numerous crevices and threads, the root form is more conducive to bacterial growth. The elimination of bacterial species may be more difficult on a root-form specimen because of reduced laser access [36]. Hakki evaluated failed threaded implants using SEM based treated with various methods. It was shown that Er:YAG was the most effective for decontaminating titanium surfaces [37]. Other studies [29, 31, 38, 39], similar to ours, have reported the efficacy of Er:YAG on flat titanium discs. Schwarz et al. studied titanium discs placed in subjects' mouths for 24 h [30]. They observed that Er:YAG was more effective than ultrasonic devices or plastic cures at removing biofilms from SLA titanium implants. Sigrun et al. reported that Er:YAG application resulted in significantly better biofilm removal from titanium surfaces than three other treatments, hand currettage, PDT, and their combinations [38]. In addition, based on the fact that there is no consensus on the optimal energy output of the laser [29], the 50 mJ/p used in this study mainly followed the study of the bactericidal effect of erbium laser by Park et al. [39].

In this study, the antimicrobial agent combined with PDT was more effective in reducing titanium biofilm vitality than 0.2% CHX (*P* < 0.05), which differs from previous reports [40]. However, because these studies differed in the light source, the photosensitizer, and the bacterial species, they cannot be compared directly. For example, Bombeccari et al. used photosensitizer (PS) of TBO and 810-nm laser radiation. They stated that using the PDT method had yielded no results [41], while the peak absorption of TBO occured at a wavelength of 635 nm [22]. Voos et al. found that PDT caused a significantly higher bacterial killing than treatment with 0.2% CHX in the ex *vivo* biofilm model (24-h biofilm) [42]. The binding of CHX to the bacterial cell wall may increase the permeability of the photosensitizer, enhancing the antibacterial effect of PDT. In this study, 0.2% CHX was used and was less effective as an antibacterial agent than photodynamic and erbium lasers. In contrast, Saffarpour found that 2% CHX had a better antibacterial effect than the Er:YAG and PDT [43]. However, high concentrations of CHX may have cytotoxic effects [44]. Therefore, further experiments are needed to determine the effective antibacterial concentration with the least toxicity.

Our study did not assess surface alterations of titanium discs after treatments. Saffarpour et al. demonstrated that the combination of Er:YAG laser irradiation, PDT with 630 nm light-emitting diodes, and TBO as a photosensitizer have strong bactericidal capacity without causing changes in the structural morphology of the implant surface [43]. Another possible source of variation between studies is the duration of treatment. In the present study, each treatment was standardized at 60 s.

The in vitro portion of the study was randomized and standardized in order to replicate clinical conditions as closely as possible. As a result, these findings may provide insight into the outcomes expected from each treatment. Further research is required in order to draw firm conclusions and develop clinical protocols.

## Conclusion

Bacterial biofilm vitality on titanium discs was reduced after Er:YAG, PDT, and CHX treatment. From most effective to least effective were Er:YAG, PDT, and CHX. All the above results were statistically significant. Furthermore, there were statistically significant differences between the three groups.

## Data Availability

The complete data and materials described in the research article are freely available from the corresponding author on reasonable request.

## Abbreviations

Er:YAG: Erbium-doped yttrium–aluminum–garnet
PDT: Photodynamic therapy
CHX: Chlorhexidine
TBO: Toluidine blue
CLSM: Confocal laser scanning microscopy
CFU: Colony-forming unit

## References

[1] French D, Ofec R, Levin L (2021). Long term clinical performance of 10 871 dental implants with up to 22 years of follow-up: a cohort study in 4247 patients. Clin Implant Dent Relat Res.

[2] Renvert S, Persson GR, Pirih FQ, Camargo PM (2018). Peri-implant health, peri-implant mucositis, and peri-implantitis: case definitions and diagnostic considerations. J Clin Periodontol.

[3] Leonhardt A, Renvert S, Dahlen G (1999). Microbial findings at failing implants. Clin Oral Implants Res.

[4] Staubli N, Walter C, Schmidt JC, Weiger R, Zitzmann NU (2017). Excess cement and the risk of peri-implant disease—a systematic review. Clin Oral Implants Res.

[5] Barbagallo G, Santagati M, Guni A, Torrisi P, Spitale A, Stefani S, Ferlito S, Nibali L (2022). Microbiome differences in periodontal, peri-implant, and healthy sites: a cross-sectional pilot study. Clin Oral Investig.

[6] Pontoriero R, Tonelli MP, Carnevale G, Mombelli A, Nyman SR, Lang NP (1994). Experimentally induced peri-implant mucositis. A clinical study in humans. Clin Oral Implants Res.

[7] Kormas I, Pedercini C, Pedercini A, Raptopoulos M, Alassy H, Wolff LF (2020). Peri-implant diseases: diagnosis, clinical, histological, microbiological characteristics and treatment strategies. A narrative review. Antibiotics (Basel).

[8] de Tapia B, Valles C, Ribeiro-Amaral T, Mor C, Herrera D, Sanz M, Nart J (2019). The adjunctive effect of a titanium brush in implant surface decontamination at peri-implantitis surgical regenerative interventions: a randomized controlled clinical trial. J Clin Periodontol.

[9] Nart J, Pons R, Valles C, Esmatges A, Sanz-Martin I, Monje A (2020). Non-surgical therapeutic outcomes of peri-implantitis: 12-month results. Clin Oral Investig.

[10] Gumus KC, Ustaoglu G, Kara L, Ercan E, Albayrak O, Tunali M (2020). Nano-hydroxyapatite airborne-particle abrasion system as an alternative surface treatment method on intraorally contaminated titanium discs. Int J Periodontics Restor Dent.

[11] Schlee M, Rathe F, Brodbeck U, Ratka C, Weigl P, Zipprich H (2019). Treatment of peri-implantitis-electrolytic cleaning versus mechanical and electrolytic cleaning-a randomized controlled clinical trial-six-month results. J Clin Med.

[12] Aoki A, Sasaki KM, Watanabe H, Ishikawa I (2000). Lasers in nonsurgical periodontal therapy. Periodontol.

[13] Drago L, Bortolin M, De Vecchi E, Agrappi S, Weinstein RL, Mattina R, Francetti L (2016). Antibiofilm activity of sandblasted and laser-modified titanium against microorganisms isolated from peri-implantitis lesions. J Chemother.

[14] Schwarz F, Bieling K, Nuesry E, Sculean A, Becker J (2006). Clinical and histological healing pattern of peri-implantitis lesions following non-surgical treatment with an Er:YAG laser. Lasers Surg Med.

[15] Polak D, Shani-Kdoshim S, Alias M, Shapira L, Stabholz A (2022). The in vitro efficacy of biofilm removal from titanium surfaces using Er:YAG laser: comparison of treatment protocols and ablation parameters. J Periodontol.

[16] Schwarz F, Jepsen S, Herten M, Sager M, Rothamel D, Becker J (2006). Influence of different treatment approaches on non-submerged and submerged healing of ligature induced peri-implantitis lesions: an experimental study in dogs. J Clin Periodontol.

[17] Nevins M, Nevins ML, Yamamoto A, Yoshino T, Ono Y, Wang CW, Kim DM (2014). Use of Er:YAG laser to decontaminate infected dental implant surface in preparation for reestablishment of bone-to-implant contact. Int J Periodontics Restor Dent.

[18] Banci HA, Strazzi-Sahyon HB, Duarte M, Cintra L, Gomes-Filho JE, Chalub LO, Berton SA, de Oliveira V, Dos Santos PH, Sivieri-Araujo G (2020). Influence of photodynamic therapy on bond strength and adhesive interface morphology of MTA based root canal sealer to different thirds of intraradicular dentin. Photodiagnosis Photodyn Ther.

[19] Chambrone L, Wang HL, Romanos GE (2018). Antimicrobial photodynamic therapy for the treatment of periodontitis and peri-implantitis: an American Academy of Periodontology best evidence review. J Periodontol.

[20] Gursoy H, Ozcakir-Tomruk C, Tanalp J, Yilmaz S (2013). Photodynamic therapy in dentistry: a literature review. Clin Oral Investig.

[21] Takasaki AA, Aoki A, Mizutani K, Schwarz F, Sculean A, Wang CY, Koshy G, Romanos G, Ishikawa I, Izumi Y (2000). Application of antimicrobial photodynamic therapy in periodontal and peri-implant diseases. Periodontol.

[22] Chiniforush N, Pourhajibagher M, Parker S, Benedicenti S, Shahabi S, Bahador A (2018). The effect of sublethal photodynamic therapy on the expression of Enterococcal surface protein (esp) encoding gene in *Enterococcus faecalis*: quantitative real-time PCR assessment. Photodiagnosis Photodyn Ther.

[23] Kadkhoda Z, Amarlu Z, Eshraghi S, Samiei N (2016). Antimicrobial effect of chlorhexidine on *Aggregatibacter actinomycetemcomitans* biofilms associated with peri-implantitis. J Dent Res Dent Clin Dent Prospects.

[24] McKenna DF, Borzabadi-Farahani A, Lynch E (2013). The effect of subgingival ozone and/or hydrogen peroxide on the development of peri-implant mucositis: a double-blind randomized controlled trial. Int J Oral Maxillofac Implants.

[25] Poppolo Deus F, Ouanounou A (2022). Chlorhexidine in dentistry: pharmacology, uses, and adverse effects. Int Dent J.

[26] Chapple ILC, Mealey BL, Van Dyke TE, Bartold PM, Dommisch H, Eickholz P, Geisinger ML, Genco RJ, Glogauer M, Goldstein M (2018). Periodontal health and gingival diseases and conditions on an intact and a reduced periodontium: consensus report of workgroup 1 of the 2017 World Workshop on the Classification of Periodontal and Peri-Implant Diseases and Conditions. J Clin Periodontol.

[27] John G, Becker J, Schwarz F (2014). Rotating titanium brush for plaque removal from rough titanium surfaces–an in vitro study. Clin Oral Implants Res.

[28] Etemadi A, Eftekhari Bayati S, Pourhajibagher M, Chiniforush N (2020). In vitro effect of antimicrobial photodynamic therapy with phycocyanin on *Aggregatibacter actinomycetemcomitans* biofilm on SLA titanium discs. Photodiagnosis Photodyn Ther.

[29] AlMoharib HS, Steffensen B, Zoukhri D, Finkelman M, Gyurko R (2021). Efficacy of an Er:YAG laser in the decontamination of dental implant surfaces: an in vitro study. J Periodontol.

[30] Desch A, Freifrau von Maltzahn N, Stumpp N, Dalton M, Yang I, Stiesch M (2020). Biofilm formation on zirconia and titanium over time-an in vivo model study. Clin Oral Implants Res.

[31] Schwarz F, Sculean A, Romanos G, Herten M, Horn N, Scherbaum W, Becker J (2005). Influence of different treatment approaches on the removal of early plaque biofilms and the viability of SAOS2 osteoblasts grown on titanium implants. Clin Oral Investig.

[32] Otsuki M, Wada M, Yamaguchi M, Kawabata S, Maeda Y, Ikebe K (2020). Evaluation of decontamination methods of oral biofilms formed on screw-shaped, rough and machined surface implants: an ex vivo study. Int J Implant Dent.

[33] Birang E, Birang R, Narimani T, Tolouei A, Fekrazad R (2019). Investigation of the antibacterial effect of laser irradiation and chemical agent on human oral biofilms contaminated titanium discs. Photodiagnosis Photodyn Ther.

[34] Wilson C, Lukowicz R, Merchant S, Valquier-Flynn H, Caballero J, Sandoval J, Okuom M, Huber C, Brooks TD, Wilson E et al. Quantitative and qualitative assessment methods for biofilm growth: a mini-review. Res Rev J Eng Technol. 2017;6(4):1-42. PMC6133255 30214915

[35] Jun HK, Jung YJ, Choi BK (2017). *Treponema denticola*, *Porphyromonas gingivalis*, and *Tannerella forsythia* induce cell death and release of endogenous danger signals. Arch Oral Biol.

[36] Kamel MS, Khosa A, Tawse-Smith A, Leichter J (2014). The use of laser therapy for dental implant surface decontamination: a narrative review of in vitro studies. Lasers Med Sci.

[37] Hakki SS, Tatar G, Dundar N, Demiralp B (2017). The effect of different cleaning methods on the surface and temperature of failed titanium implants: an in vitro study. Lasers Med Sci.

[38] Eick S, Meier I, Spoerle F, Bender P, Aoki A, Izumi Y, Salvi GE, Sculean A (2017). In vitro-activity of Er:Yag laser in comparison with other treatment modalities on biofilm ablation from implant and tooth surfaces. PLoS ONE.

[39] Park SH, Kim OJ, Chung HJ, Kim OS (2020). Effect of a Er, Cr:YSGG laser and a Er:YAG laser treatment on oral biofilm-contaminated titanium. J Appl Oral Sci.

[40] Sculean A, Schwarz F, Becker J (2005). Anti-infective therapy with an Er:YAG laser: influence on peri-implant healing. Expert Rev Med Devices.

[41] Bombeccari GP, Guzzi G, Gualini F, Gualini S, Santoro F, Spadari F (2013). Photodynamic therapy to treat periimplantitis. Implant Dent.

[42] Voos AC, Kranz S, Tonndorf-Martini S, Voelpel A, Sigusch H, Staudte H, Albrecht V, Sigusch BW (2014). Photodynamic antimicrobial effect of safranine O on an ex vivo periodontal biofilm. Lasers Surg Med.

[43] Saffarpour A, Nozari A, Fekrazad R, Saffarpour A, Heibati MN, Iranparvar K (2018). Microstructural evaluation of contaminated implant surface treated by laser, photodynamic therapy, and chlorhexidine 2 percent. Int J Oral Maxillofac Implants.

[44] Wyganowska-Swiatkowska M, Kotwicka M, Urbaniak P, Nowak A, Skrzypczak-Jankun E, Jankun J (2016). Clinical implications of the growth-suppressive effects of chlorhexidine at low and high concentrations on human gingival fibroblasts and changes in morphology. Int J Mol Med.

